# No difference in postoperative patient satisfaction rates between mechanical and kinematic alignment total knee arthroplasty: A systematic review

**DOI:** 10.1002/jeo2.12101

**Published:** 2024-07-24

**Authors:** Zainab‐Aqeel Khan, Alexandra Leica, Manuel‐Paul Sava, Michael T. Hirschmann

**Affiliations:** ^1^ Department of Orthopaedic Surgery and Traumatology Kantonsspital Baselland (Bruderholz, Liestal, Laufen) Bruderholz Switzerland; ^2^ Department of Clinical Research, Research Group Michael T. Hirschmann, Regenerative Medicine & Biomechanics University of Basel Basel Switzerland; ^3^ Department of Research AO Hospital Karachi Pakistan; ^4^ Department of Molecular Medicine and Surgery Stockholm Sports Trauma Research Center, Karolinska Institutet Stockholm Sweden

**Keywords:** alignment, kinematical alignment, mechanical alignment, patient satisfaction, patient‐reported outcomes, total knee arthroplasty

## Abstract

**Purpose:**

The purpose of this systematic review was to compare patient satisfaction patient‐reported outcomes (PROMs) levels after mechanically aligned (MA) and kinematically aligned (KA) total knee arthroplasty (TKA).

**Methods:**

A systematic literature search following PRISMA guidelines was conducted on PubMed, Embase, Medline and Scopus to identify potentially relevant articles for this review, published from the beginning of March 2013 until the end of October 2023. Only articles reporting satisfaction after KA TKA, MA TKA or both were included, which use valid and reliable tools for the evaluation and reporting of satisfaction after TKA. Title, authors, year of publication, study design, level of evidence, follow‐up period, patients' demographic data, sample size, type of satisfaction score, postoperative satisfaction score, postoperative alignment, statistical significance, as well as other variables, were extracted for analysis. An Agency for Healthcare Research and Quality's (AHRQ) design‐specific scale was used for assessing randomized control trials (RCTs). The nonrandomized control trials were evaluated by using the Joanna Briggs Institute's (JBI) Critical Appraisal Tool. The Newcastle‐Ottawa Scale (NOS) was also used to assess cohort studies, while case series were evaluated using the NIH Quality Assessment Tool for Case Series Studies.

**Results:**

The initial search identified 316 studies, of which 178 were considered for screening. Eleven studies completely fulfilled the inclusion criteria, including one RCT, five nonrandomized control trials/quasi‐experiments, three case series, and two cohort studies. The total number of patients recruited for MA TKA was 1740. Conversely, 497 patients were enrolled for KA TKA. Five studies used the visual analogue scale (VAS) for assessing postoperative patient satisfaction, four used the Knee Society Score (KSS) 2011 version and two Likert‐based types of scores. Overall, the highest mean satisfaction score of KSS 2011 was 31.5 ± 6.6 in the MA group, and 29.8 ± 80 in the KA group in four studies. All of them showed high postoperative patient satisfaction rates for both MA and KA TKA, but with no statistically significant difference between them (*p* > 0.05).

**Conclusion:**

Both mechanically aligned total knee arthroplasty, as well as kinematically aligned total knee arthroplasty led to high rates of postoperative patient satisfaction, with no statistically significant differences between them.

**Level of Evidence:**

Level III, systematic review.

**Abbreviations**
AHRQ: Agency for Healthcare Research and Quality
JBI: Joanna Briggs Institute
KA: kinematic alignment
KSS: knee society score
MA: mechanical alignment
NOS: Newcastle‐Ottawa Scale
PFA: patellofemoral arthroplasty
PROMs: patient‐reported outcomes
RCTs: randomized control trials
rTKA: revision total arthroplasty
TKA: total knee replacement
UKA: unicondylar knee arthroplasty
VAS: visual analogue scale

## INTRODUCTION

Postoperative restoration of a neutral limb alignment has been the primary goal of conventional total knee arthroplasty (TKA) over the past two decades [28]. The mechanical alignment (MA) philosophy emphasizes the importance of a mechanically aligned knee, as the implanted femoral and tibial TKA components should be perpendicular to the mechanical axis of the limb [28]. This, in turn, leads to a more balanced loading and thus increases components' survival [28, 36]. However, this alignment philosophy does not respect the anatomical joint line orientation and phenotypes of the native knee and, therefore, alters surrounding soft tissues' tension [36]. Additionally, MA does not restore the individual knee kinematics [12]. The rates of residual knee symptoms and patient dissatisfaction after MA TKA have been reported to be as high as 50% and 20%, respectively [23]. Furthermore, changing the native alignment could play a significant role in postoperative patient satisfaction levels and patient‐reported outcomes (PROMs) [23]. However, recently, the preferred alignment philosophy in TKA has shifted from a systematic approach toward a more personalized one in search of higher functionality and patient satisfaction [2, 16, 34]. In this regard, kinematic alignment (KA) has gained significant momentum, as it conceptually aims to restore the alignment and kinematics of the native prearthritic knee [19].

Nonetheless, given the still high dissatisfaction rate after present TKAs (10%–20%), and the current widespread use of both alignment strategies, the need to identify any possible influence of the chosen alignment strategy over patient satisfaction levels, which particularly leads to a better postoperative patient satisfaction score becomes apparent [4, 6]. To better understand the patient's perspective, the analysis of PROMs and patient satisfaction is crucial [5]. Some of the most used PROMs for achieving satisfaction after TKA are Visual Analogue Scales (VAS), the new Knee Society Knee Scoring System (KSS 2011) and Likert‐Scale [11, 15]. Therefore, the aim of this systematic review is to compare patient satisfaction PROMs after MA and KA TKA. The hypothesis of this study is that the alignment strategy does influence patient satisfaction levels after TKA and KA TKA produces superior postoperative satisfaction levels when compared to MA TKA.

## METHODS

A systematic literature search following PRISMA guidelines was conducted on PubMed, Embase, Medline and Scopus to identify potentially relevant articles for this review, published from the beginning of March 2013 until the end of October 2023. Mesh terms such as “alignment technique,” “total knee arthroplasty,” and “dissatisfaction” were used for building a search strategy in each database accordingly. A detailed description of the search strategy can be found in File S1. The study protocol was registered with PROSPERO (CRD42023492219).

Identified studies have been imported into Covidence® (Veritas Health Innovation Ltd) and removal of duplicates has been automatically performed. Two authors independently underwent title and abstract screening. The same two authors have performed full‐text analysis. In case of disagreement/uncertainty, a third author was consulted. The selection was based on the following inclusion criteria: full‐text clinical studies in English, published in peer‐reviewed journals, which use valid and reliable tools for the evaluation and reporting of satisfaction after TKA. Only articles reporting satisfaction after KA TKA (unrestricted), MA TKA or both were included. All preprints, abstract‐only studies, protocols, literature reviews, meta‐analyses, expert opinion articles, book chapters, surgical technique studies, and studies pertaining to restricted or inverse KA alignment, unicondylar knee arthroplasty (UKA), patellofemoral arthroplasty (PFA), or revision total knee arthroplasty (rTKA), were excluded. Studies with unavailable numeric data (graphical only) were also excluded.

### Data extraction

Title, authors, year of publication, study design, level of evidence, follow‐up period, patient's demographic data, sample size, type of satisfaction score, preoperative, and/or postoperative satisfaction score, postoperative alignment, statistical significance, as well as other variables, were extracted for analysis.

### Quality assessment

All included studies were assessed for their quality according to the study design. In this review, an Agency for Healthcare Research and Quality (AHRQ) design‐specific scale for randomized control trials (RCTs) [35] (Table 1). The nonrandomized control trials were assessed by using the Joanna Briggs Institute's (JBI) Critical Appraisal Tool [14] (Table 2). The Newcastle‐Ottawa Scale (NOS) was also used to assess cohort studies [30] (Table 3), and case series were evaluated by using the NIH Quality Assessment Tool [20] (Table 4).

**Table 1:** Quality assessment criteria for randomized control trials (RCTs).

Quality assessment	Blyth et al.[2]
Was the allocation sequence generated adequately?	Yes
Was the allocation of treatment adequately concealed?	Yes
Did researchers rule out any unintended exposure that might bias results?	No
Were participants analysed within the groups they were originally assigned to?	Yes
Was the length of follow‐up different between the groups?	No
Were the outcome assessors blinded to the intervention or exposure status of participants?	Yes
Were the potential outcomes prespecified by the researchers? Are all pre‐specified outcomes reported?	Yes
If attrition was a concern, were missing data handled appropriately?	Yes
Were outcomes assessed using valid and reliable measures across all study participants?	Yes
Judgement on the risk of bias?	Low risk

*Note*: Assessed using AHRQ design‐specific scale.

**Table 2:** Quality assessment criteria for nonrandomized control trials (RCTs).

Quality assessment	Khuangsirikul et al.[13]	French et al.[7]	Niki et al.[19]	Niki et al.[20]	Tsubosaka et al.[24]
Is it clear in the study what is the ‘cause’ and what is the ‘effect’?	Yes	Yes	Yes	Yes	Yes
Were the participants included in any comparisons similar?	Yes	Yes	Yes	Yes	Yes
Were the participants included in any comparisons receiving similar treatment/care, other than the exposure or intervention of interest?	No	No	No	No	No
Was there a control group?	Yes	Yes	Yes	Yes	Yes
Were there multiple measurements of the outcome, both pre and postintervention/exposure?	Yes	Yes	Yes	Yes	Yes
Was follow‐up complete, and if not, were differences between groups in terms of their follow‐up adequately described and analysed?	Yes	Yes	Yes	Yes	Yes
Were the outcomes of participants included in any comparisons measured in the same way?	Yes	Yes	Yes	Yes	Yes
Were outcomes measured in a reliable way?	Yes	Yes	Yes	Yes	Yes
Was appropriate statistical analysis used?	Yes	Yes	Yes	Yes	Yes

*Note*: The Joanna Briggs Institute Critical Appraisal Tools for Use in JBI Systematic Reviews.

**Table 3:** Quality assessment criteria for Cohort study.

Quality assessment	Aunan et al.[1]	Koh et al.[14]
Representativeness of the exposed cohort	Yes	Yes
Selection of the nonexposed cohort	Yes	Yes
Ascertainment of exposure	Yes	Yes
Demonstration that outcome of interest was not present at the start of the study	Yes	Yes
Comparability of cohorts on the basis of the design or analysis	Yes	Yes
Assessment of outcome	Yes	Yes
Was follow‐up long enough for outcomes to occur	Yes	Yes
Adequacy of follow‐up of cohorts	Yes	Yes
Total Newcastle Ottawa Scale	8	8

*Note*: The Newcastle‐Ottawa scale.

**Table 4:** Quality assessment criteria for case series.

Quality assessment	Umatani et al. [26]	Turan et al. [25]	Firer et al. [6]
Was the study question or objective clearly stated?	Yes	Yes	Yes
Was the study population clearly and fully described, including a case definition?	Yes	Yes	Yes
Were the cases consecutive?	Yes	Yes	Yes
Were the subjects comparable?	No	No	No
Was the intervention clearly described?	Yes	Yes	Yes
Were the outcome measures clearly defined, valid, reliable, and implemented consistently across all study participants?	Yes	Yes	Yes
Was the length of follow‐up adequate?	Yes	Yes	Yes
Were the statistical methods well‐described?	Yes	Yes	Yes
Were the results well‐described?	Yes	Yes	Yes

*Note*: Quality Assessment Tool for Case Series Studies.

### Statistical analysis

The heterogeneity of included studies and different tools used for outcome measures precluded a meta‐analysis. Therefore, continuous variables were defined by using descriptive statistics such as means, standard deviations and percentages. The quality assessment was performed for all the content used in this systematic review.

## RESULTS

### Search results

**Figure 1:**
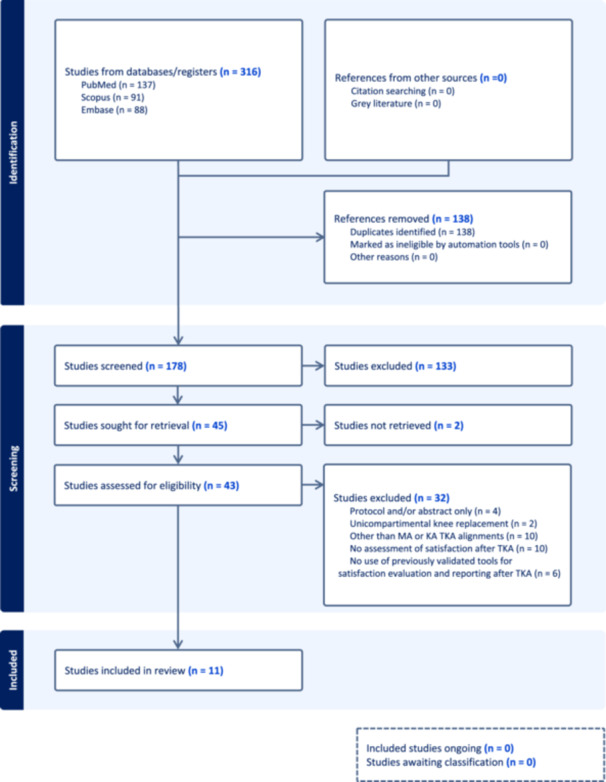
Flowchart of the study selection process according to the PRISMA 2020 statement: An updated guideline for reporting systematic reviews.

The initial search identified 316 studies, of which 178 were considered for screening. Details of the study selection and inclusion process are illustrated in Figure 1 (PRISMA Flowchart). Eleven studies completely fulfilled the inclusion criteria [1, 3, 7, 8, 17, 18, 24, 25, 31, 32, 33], including one RCT [3], five nonrandomized control trials/quasi‐experiments [3, 8, 24, 25, 31], three case series [7, 32, 33], and two cohort studies [1, 18]. Three studies compared satisfaction outcomes after TKA between MA TKA and KA TKA [18, 24, 25], three reported satisfaction outcomes after KA TKA only [31, 32] and five reported satisfaction outcomes after MA TKA only [1, 3, 6, 7, 17, 33]. The total number of patients recruited for MA TKA throughout the included studies was 1740. Conversely, 497 patients were enrolled for KA TKA. Patients' baseline characteristics were comparable between studies (Table 5).

**Table 5:** Overview of selected studies.

Authors	Year of publication	Study design	Evidence level	Follow‐up (months)	Sample size (patients)	Age ([years mean/median ± SD/limits])	Gender ([males] %)
Blyth et al.[17]	2014	Randomized control trial	I	12	Navigated TKA: 101 Conventional TKA: 97	Navigated TKA: 65.6 (43–85) Conventional TKA: 65.4 (42–85)	MA: 41.41% (82)
Khuangsirikul et al.[18]	2016	Nonrandomized control trial	III	CAS TKA: 120.57 Conventional TKA: 120.15	CAS TKA: 70 Conventional TKA: 74	CAS TKA: 77.10 ± 6.69 Conventional TKA: 76.73 ± 8.02	CAS TKA: 10%(10) Conventional TKA: 9.46% (7)
Umatani et al.[19]	2021	Retrospective Case series	IV	12	MA: 117	MA: 75.6 ± 6.7 (55 to 91)	MA: 19.65% (23)
Turan et al.[20]	2022	Retrospective case‐series	IV	12	KA: 109	KA: 66.3 ± 9.3 (34–89)	KA: 15.59% (17)
Aunan et al.[21]	2018	Prospective cohort study	II	36	MA: 129^a^ With ligament balancing: Without ligament balancing:	With ligament balancing: 69 (42–81) Without ligament balancing: 70 (53–82)	With ligament balancing: 27.9%(36) Without ligamen tbalancing: 15.50%(20)
French et al.[22]	2019	Nonrandomized control trial	III	MS: 13.1 (10.3–18.4) CR: 13.7 (11.0–18.9)	MS: 46 CR: 44	MS: 69.5 (6.9) (53–86) CR: 66.1 (7.9) (47–80)	MS: 34.78% (16) CR: 50% (22)
Koh et al.[23]	2020	Retrospective Cohort Study	III	24	MA: 93 KA: 93	MA: 67 (7.4) KA: 69.1 (8.1)	MA: 21.50% (20) KA: 24.73%(23)
Niki et al.[24]	2018	Nonrandomized control trial	III	31.6	MA: 45 KA: 45	MA: 72.8KA: 70.4	MA: 22.2% (10) KA: 28.9% (13)
Niki et al.[25]	2019	Nonrandomized control trial	III	24	MA: 100 KA: 100	MA: 74.2 ± 7.9KA: 73.4 ± 8.5	MA: 15% (15) KA: 25% (25)
Tsubosaka et al.[26]	2019	Nonrandomized control trial	III	12	OrthoPilot: 30 iASSIST: 30	OrthoPilot: 74.2 ± 9.4 (55 to 84)iASSIST: 75.8 ± 7.0 (61 to 85)	OrthoPilot: 16.55% (5) iASSIST: 26.66% (8)
Firer et al.[27]	2018	Case series	IV	51.6	MA: 914	MA: 67 (47–94)	MA: 46% (420)

Abbreviations: CAS, computer‐assisted surgery; CR, cruciate‐retaining; KA, kinematical alignment; MA, mechanical alignment; MIS group, minimal invasive surgery group; MS, medially stabilized; SD, standard deviation; TKA, total knee arthroplasty.

a Knees.

Four studies used 2011 KSS for assessing satisfaction [24, 25, 31, 33], while five studies have used VAS [1, 3, 7, 8, 32]. In all the included studies, the method of satisfaction assessment was clearly mentioned in the methodology section. All details regarding the use of satisfaction assessment tools are presented in Table 6.

**Table 6:** Overview of used instruments for satisfaction assessment.

Authors	Postoperative alignment	Type of satisfaction score	MA preoperative values	KA preoperative values	*p*‐Value	MA postoperative values	KA postoperative values	*p*‐Value
Blyth et al.[17]	MA	VAS^a^	‐	‐	‐	Navigated TKA: Very satisfied 64% Satisfied 26% Conventional TKA: Very satisfied 56% Satisfied 30%	‐	0.34
Khuangsirikul et al.[18]	MA	Self‐administered patient satisfaction postoperative	‐	‐	‐	Conventional TKA: 100 (35.7–100) CAS TKA: 100 (25–100)	‐	0.889
Umatani et al.[19]	MA	KSS 2011	13.9 ± 6.1 (0–30)	‐	‐	25.6 ± 8.0 (6–40)	‐	0.001
Turan et al.[20]	KA	VAS	‐	‐	‐	‐	8.3 ± 1.5	N/A
Aunan et al.[21]	MA	VAS^d^	‐	‐	‐	With ligament balancing: 98 (10) Without ligament balancing: 98 (10)	‐	0.6
French et al.[22]	KA	VAS	‐	‐	‐	‐	CR9.1 (0.9) MS9.3 (1.2)	0.314
Koh et al.[23]	KA and MA	LikertScale^b^	‐	‐	‐	97.8%	90.3%	n.s.
Niki et al.[24]	KA and MA	KSS 2011	12.6 ± 6.0	12.4 ± 4.2	n.s.	31.5 ± 6.6	29.8 ± 8.0	n.s.
Niki et al.[25]	KA and MA	KSS 2011	‐	‐	‐	28.9 ± 8.6	27.4 ± 7.2	n.s.
Tsubosaka et al.[26]	KA	KSS 2011	‐	OrthoPilot: 13.7 ± 6.7 (0–24)ASSIST: 12.7 ± 7.3 (0–30)	0.5881	‐	OrthoPilot:28.4 ± 7.0 (14‐40) ASSIST: 28.5 ± 7.1 (16‐40)	0.9768
Firer et al.[27]	MA	VAS^c^	‐	‐	‐	Satisfied patients: 9.53 (7.3–10.0) Dissatisfied patients: 3.78 (0.0–6.3)	‐	<0.05

Abbreviations: CAS, computer‐assisted surgery; CR, cruciate‐retaining; KA, kinematical alignment; KSS, knee society score; MA, mechanical alignment; MIS group, minimal invasive surgery group; MS, medially stabilized; N/A, not applicable; Ns, not significant; TKA, total knee arthroplasty; VAS, visual analogue scale‐satisfaction.

a % grade satisfaction in percentages.

b Likert scale adapted from North American Spine Society Questionnaire. Likert scale of 1‐6, with a score of 1 representing “Excellent satisfaction” and a score of 6 representing “Extreme dissatisfaction”. 1–3 “satisfied” and 4–6 “dissatisfied.”

c VAS (0—worst − 10—best).

d VAS (0—worst − 100—best).

### Association between satisfaction score and alignment target

Out of all included studies, only three mentioned the preoperative satisfaction score [24, 31, 33] (Table 6). Of the four studies, which used KSS 2011, two compared satisfaction between MA and KA, while the other two reported satisfaction in only MA or KA groups individually. Overall, the highest mean satisfaction score of KSS 2011 was 31.5 ± 6.6 in the MA group, and 29.8 ± 80 in the KA group [24, 25, 31, 32]. The two studies, which compared satisfaction scores in KSS 2011 between MA and KA, did not report any statistically significant differences between the groups [24, 25].

Five studies which used VAS reported satisfaction mean/median and standard deviation/interquartile range, except Blyth et al. [3]. Among these five studies, three reported these outcomes after MA TKA [1, 7, 14] and two after KA TKA [8, 32]. The reported results indicate that the satisfaction scores after MA and KA TKA are equally high, displaying no relation to the chosen alignment technique.

Additionally, Khuangsirikul et al. [17] evaluated patient satisfaction after MA TKA by using a self‐administered patient satisfaction score. The overall satisfaction rate was almost 100%. Lastly, Koh et al. [18] reported patient satisfaction levels on a Likert‐type scale adapted from the North American Spine Society Questionnaire. It showed 97.8% satisfied patients after MA TKA and just 90.3% after KA TKA. The findings did not, however, qualify as a statistically significant difference.

## DISCUSSION

The most important findings of the present review were that both MA and KA TKA led to high postoperative patient satisfaction levels and that although the heterogeneity in used assessment tools was quite high, no statistically significant difference in patient satisfaction when comparing KA with MA TKA was observed.

The general idea, in the current available literature mentions satisfaction rates from 80% to 100% after TKA procedures. However, the number of systematic reviews and/or meta‐analyses focusing on comparing satisfaction scores between alignments and providing specific findings is rather low. The vast majority of studies are focused on reporting outcomes through compounded functional scores, which contain satisfaction subcomponents, but to a lesser extent, and more often than not, do not thoroughly explore the satisfaction aspect. Additionally, the presented data is also quite heterogeneous. In a recent meta‐analysis, which focused on comparing multiple postoperative outcomes, it has been indicated that there is no significant difference in any outcome measure, including satisfaction scores, between KA and MA TKA [29]. In contrast, Liu et al., who also conducted a recent meta‐analysis, in which functional and clinical postoperative outcomes are being compared between KA and MA TKA, presented superior functional outcomes after KA in comparison to MA but with identical clinical outcomes [20]. However, again, the satisfaction component has been underexplored, being just a subcomponent of a compounded functional score. Gao et al. and Luo et al., both conducting systematic reviews, focusing on postoperative outcomes comparison between MA and KA TKA, have found no statistically significant differences between patients' quality of life after MA and KA TKA [9, 22]. However, with no reference to the satisfaction subcomponent. Therefore, due to this high variability in reporting satisfaction after TKA, through different scores, some of them not validated for satisfaction assessment, it is challenging to compare our findings with the ones previously presented in the literature.

Nonetheless, there are certain significant limitations to this systematic review. Firstly, the quality of the included studies is moderately low, as most of them are level III and IV studies. This review unrestricted KA to maintain homogeneity between groups. This may compromise the assessment of satisfaction for all types of KA TKA (restricted/inverse), which may impact the overall patient satisfaction. The comparison of patient satisfaction between two alignments was made for different implants due to heterogeneity in the published literature. Therefore, it was impossible to control confounding factors like differences in implant and post‐operative rehabilitation. Furthermore, the reporting of satisfaction after TKA was highly variable in all studies, regardless to alignment, multiple scores and methodologies being used for patient satisfaction assessment. Therefore, performing a meta‐analysis of the data for more concrete findings was not possible. Moreover, this review included studies from different cultural backgrounds. A previously published report indicated that cultural differences in the healthcare system might influence patient‐reported satisfaction [14]. This might also impact the comparative measures of satisfaction between all groups. However, considering the aim of the review, it was determined that it would be useful to evaluate the complete body of evidence published in the selected period, rather than to include only one particular geographic area. Nevertheless, further high quality level studies are required to more accurately and clearly assess and compare postoperative patient satisfaction between MA and KA TKA. Therefore, future efforts should be made to unify the reporting and assessing postoperative patient satisfaction methods after TKA through the use of dedicated and validated instruments. The validation of patient‐centred instruments to measure satisfaction after TKA would not only improve the quality and assessment of satisfaction but also facilitate the comparison between alignments.

However, for this purpose, clinical studies need to include detailed information on knee phenotypes as it can be expected that some phenotypes are better off with different alignment philosophies and some are not [10, 21, 26, 27].

## CONCLUSION

Both mechanically aligned TKA, as well as kinematically aligned TKA led to high rates of postoperative patient satisfaction, but with no statistically significant differences between them.

## AUTHOR CONTRIBUTIONS

*Study conception and design and supervise the project*: Michael T. Hirschmann. *Conduct a search, review of the literature, data synthesis and manuscript preparation*: Zainab‐Aqeel Khan. *Assessing the quality of the studies*: Alexandra Leica and Manuel Sava. All authors reviewed the results and approved the final version of the manuscript.

## CONFLICT OF INTEREST STATEMENT

The authors declare no conflict of interest.

## ETHICS STATEMENT

Not applicable.

## Supporting information

Supporting information.

## Data Availability

Data are available at request from the hospital suppository.
