# Supplementary material for: No difference in postoperative patient satisfaction rates between mechanical and kinematic alignment total knee arthroplasty: A systematic review
Source: J Exp Orthop. 2024 Jul 24;11(3):e12101. doi: 10.1002/jeo2.12101 (PMC11267171; doi:10.1002/jeo2.12101)
Supplement: Supplementary file 1 — Supporting information. [file JEO2-11-e12101-s001.docx]

**Detailed Search Strategy**

***Pubmed***

(("dissatisfaction"[Text Word] OR "patient satisfaction"[Text Word] OR "unhappy patients"[Text Word] OR "satisfaction"[Text Word] OR "Patient Preferences"[Text Word] OR "Patient reported outcomes"[Text Word] OR "patient satisfaction"[MeSH Major Topic] OR "patient satisfaction/statistics and numerical data"[MeSH Major Topic]) AND "2013/03/01 00:00":"3000/01/01 05:00"[Date - Publication] AND (("total knee replacement"[Text Word] OR "total knee arthroplasty"[Text Word] OR "knee replacement"[Text Word] OR "Knee arthroplasty"[Text Word] OR "TKR"[Text Word] OR "TKA"[Text Word] OR "arthroplasty, replacement, knee"[MeSH Major Topic] OR "arthroplasty, replacement, knee/methods"[MeSH Major Topic] OR "knee joint/surgery"[MeSH Terms] OR "Knee Prosthesis"[MeSH Terms] OR "Knee Prosthesis"[MeSH Major Topic] OR "arthroplasty, replacement, knee"[MeSH Major Topic] OR "Knee Joint"[MeSH Terms]) AND "2013/03/01 00:00":"3000/01/01 05:00"[Date - Publication]) AND (("knee alignment"[Text Word] OR "mechanical alignment"[Text Word] OR "kinematic alignment"[Text Word] OR "kinematical alignment"[Text Word] OR "KA"[Text Word] OR "MA"[Text Word]) AND "2013/03/01 00:00":"3000/01/01 05:00"[Date - Publication])) AND (y_10[Filter])

***Scopus***

(TITLE-ABS-KEY ( "Total knee replacement" )  OR  TITLE-ABS-KEY ( "Total knee arthroplasty" )  OR  TITLE-ABS-KEY ( TKA )  OR  TITLE-ABS-KEY ( TKR )  AND  TITLE-ABS-KEY ( "kinematic alignment" )  OR  TITLE-ABS-KEY ( "mechanical alignment" )  AND  TITLE-ABS-KEY ( "patient satisfaction" )  OR  TITLE-ABS-KEY ( dissatisfaction ) )

***Embase***

('total knee arthroplasty'/exp OR 'total knee arthroplasty') AND ('mechanical alignment' OR 'kinematic alignment') AND ('satisfaction' OR ‘dissatisfaction’)
